# Prevalence of infant bronchiolitis‐coded healthcare encounters attributable to RSV

**DOI:** 10.1002/hsr2.91

**Published:** 2018-10-12

**Authors:** Kedir N. Turi, Pingsheng Wu, Gabriel J. Escobar, Tebeb Gebretsadik, Tan Ding, Eileen M. Walsh, Sherian X. Li, Kecia N. Carroll, Tina V. Hartert

**Affiliations:** ^1^ Department of Medicine Vanderbilt University Medical Center Nashville TN USA; ^2^ Department of Biostatistics Vanderbilt University Medical Center Nashville TN USA; ^3^ Kaiser Permanente Northern California, Systems Research Initiative, Perinatal Research Unit, Division of Research Kaiser Permanente Oakland CA USA; ^4^ Department of Inpatient Pediatrics Kaiser Permanente Medical Center Walnut Creek CA USA; ^5^ Department of Pediatrics Vanderbilt University Medical Center Nashville TN USA

**Keywords:** bronchiolitis, ICD‐9‐CM, infant, respiratory syncytial virus (RSV)

## Abstract

**Aim:**

We sought to determine the proportion of bronchiolitis episodes attributable to respiratory syncytial virus (RSV) among ICD‐9 coded infant bronchiolitis episodes which were tested for RSV.

**Methods:**

Bronchiolitis healthcare encounters were extracted from Kaiser Permanente Northern California databases for years 2006 to 2009. We used ICD‐9 codes for bronchiolitis to capture bronchiolitis‐related healthcare encounters including hospital admissions (Hospitalization), emergency department visits (EDV), and outpatient visits (OPV). We reported the monthly proportion of RSV‐positive bronchiolitis episodes among tested bronchiolitis episodes. We used logistic regression to assess association between bronchiolitis episodes and patient demographic and health care characteristics. We also used logistic regression to assess association between decision to test and patient demographics and health care characteristics.

**Results:**

Among 10,411 ICD‐9 coded infant bronchiolitis episodes, 29% were RSV tested. Fifty one percent of those tested were RSV positive. Between December and February, and in infants ≤6 months, the proportion of bronchiolitis episodes that were attributable to RSV was 77.2% among hospitalized episodes, 78.3% among EDV episodes, and 60.9% among OPV episodes, respectively. The proportion of RSV‐positive bronchiolitis episodes varied based upon infant age at diagnosis, level of health care service used, and time of the year of the episode.

**Conclusion:**

Estimation of the proportion of ICD‐9 coded bronchiolitis episodes attributable to RSV is more specific when restricting to bronchiolitis episodes during peak months, younger infant age, and those requiring higher level of healthcare.

## Abstract 2

Perspective on this article: Is it RSV?

**Abbreviations**
AAP: American Academy of Pediatrics
CI: Confidence Interval
EDV: Emergency Department Visit
ICD‐9: International classification of diseases
KPNC: Kaiser Permanente Northern California
OPV: Outpatient Visit
PCR: Polymerase Chain Reaction
PRIMA: *Prevention of RSV: Impact on Morbidity and Asthma*
RSV: Respiratory syncytial virus

## 1 INTRODUCTION

Bronchiolitis is a lower respiratory tract infection characterized by increased respiratory effort and wheezing.^1^ Bronchiolitis in infants (defined as age ≤ 12 months) is most often associated with respiratory syncytial virus (RSV), and is a major cause of hospitalization during infancy.^2^ However, not all bronchiolitis is caused by RSV. Reports of prevalence and rates of RSV‐bronchiolitis health care use are often based on the International Classification of Disease (ICD) codes.^3^, ^4^, ^5^ However, ICD codes do not always overlap with other means of classification, including clinical laboratory results.^6^, ^7^, ^8^ Consequently, the reported incidence, proportion, prevalence, and trend of RSV‐bronchiolitis in healthcare encounters likely include misclassification of episodes.

The purpose of this study was to calculate the proportion of RSV‐positive bronchiolitis episodes among infant bronchiolitis episodes identified by ICD‐9 codes, utilizing a population of infants in whom both ICD‐9 codes as well as laboratory RSV detection methods, were available in electronic health records. Improving the specificity of ICD‐coded RSV‐bronchiolitis episodes may help surveillance and prevention strategies by identifying RSV peak season and target age groups.

## 2 MATERIALS AND METHODS

This study was approved by both the Kaiser Permanente Northern California (KPNC) and Vanderbilt University Institutional Review Boards. Informed consent process was waived in accordance with 45 CFR 46.116 (d) by Vanderbilt University Institutional Review Board. Use of birth certificate data was approved by the State of California Committee for the Protection of Human Subjects.

Infant (defined as age ≤ 12 months) bronchiolitis healthcare encounters were extracted from KPNC databases for the years 2006 to 2009, a subset of the “Prevention of RSV: Impact on Morbidity and Asthma (PRIMA)” cohort study.^9^ KPNC database is an electronic health record (EHR) of an integrated healthcare system such that all care is provided in a closed system and documented in an EHR. The database, which includes electronic health records as well as corresponding ICD‐9 codes, has been built for research purposes. We used ICD‐9 codes (466.11 for RSV‐bronchiolitis; 480.1 for RSV‐pneumonia; and 466.19 for non‐RSV bronchiolitis) to capture bronchiolitis‐related healthcare encounters (we refer to these as ‘bronchiolitis episodes’ throughout the manuscript, see Table S1 for a detailed explanation of ICD‐9 codes used). The KPNC database also includes the level of healthcare services received for the bronchiolitis episodes, including hospital admissions (Hospitalization), emergency department visits (EDV), and outpatient visits (OPV). The World Health Organization developed ICD‐9 codes, and the United States uses modified ICD‐9 diagnosis codes to identify diseases and health conditions across all claims. ICD‐9 codes were in use before converting to ICD‐10 version in 2015; while the codes for bronchiolitis have changed, their descriptors have not.

Encounters that occurred within 14 days of each other were considered as one episode and were classified based on the highest level of care visit type the infant experienced (Hospitalization > EDV > OPV), while the bronchiolitis encounters that were 14 days or more apart were treated as independent episodes. More than one test and/or one detection method (antigen, culture, and Polymerase Chain Reaction [PCR]) could be performed during one bronchiolitis episode. The episode was classified as RSV positive if any of the tests were positive. If multiple tests per episode were negative, only the PCR result was retained. Since 2006, KPNC started using primarily PCR to test for RSV (78.3%), with antigen and culture methods constituting only 13.2% and 8.5% of the tests, respectively. In this study, we included ICD‐9^10^‐identified infant bronchiolitis episodes captured in the PRIMA cohort from 2006 to 2009 and which were also tested for RSV.

We reported the monthly proportion of RSV‐positive bronchiolitis episodes among tested bronchiolitis episodes. The proportion of RSV‐positive bronchiolitis episodes was further calculated among the subset of tested bronchiolitis episodes: by infant age at the time of bronchiolitis diagnosis (≤6 months and ≤ 3 months), by both infant age at the time of bronchiolitis diagnosis (≤6 months) and the severity of the bronchiolitis episodes (OPV, EDV, and Hospitalization), and by gestational age (term vs. preterm [gestational age < 37 weeks]). We conducted post hoc analysis using multivariable logistic regression to assess the association of infant age at bronchiolitis diagnosis, sex, season of the episode (peak RSV season vs. non‐peak season), severity of the bronchiolitis episodes (OPV, EDV, and Hospitalization), RSV‐immunoprophylaxis eligibility based on American Academy of Pediatrics (AAP) guidelines,^11^, ^12^ and RSV‐immunoprophylaxis receipt (ever receipt and never receipt) with outcome of RSV positivity. Lastly, we used multivariable logistic regression to identify factors that were associated with testing for RSV. Analyses were conducted using Stata software version 15 (College Station, TX).^13^

## 3 RESULTS

A total of 10,411 ICD‐9 coded bronchiolitis episodes among 6,655 infants were identified over the 4‐year period, 2006 to 2009. These included: 5.8% (*n* = 608) ICD‐9 code 466.11 (RSV‐bronchiolitis), 0.2% (*n* = 25) ICD‐9 code 480.1 (RSV‐pneumonia), and 93.9% (*n* = 9,778) ICD‐9 code 466.19 (non‐RSV bronchiolitis). Among the 10,411 ICD‐9 coded bronchiolitis episodes, the median infant age at the first bronchiolitis episode was 184 days (interquartile range, IQR: 120, 252). Sixty one percent of episodes (6,381 episodes among 3,964 infants) were in male infants, and 13.8% (1,440 episodes among 820 infants) were among preterm infants. Of total bronchiolitis episodes, 89.0% (*n* = 9,269), 4.1% (*n* = 425), and 6.9% (*n* = 717) were OPV, EDV, and hospitalization, respectively.

In total, 29.2% (*n* = 3,040 episodes among 2,057 infants) of all ICD‐9 identified bronchiolitis episodes during infancy were laboratory tested for RSV. The proportion of bronchiolitis episodes that were tested within each of the three ICD‐9 codes was as follows: among 608 RSV‐bronchiolitis coded episodes, 59.4% were tested; among 25 RSV‐pneumonia coded episodes, 44.0% were tested; and among 9,778 non‐RSV coded bronchiolitis episodes, 27.3% were tested (Table 1). Compared with the first bronchiolitis episodes that were not tested, first episodes that were tested for RSV were more likely to be among younger infants (164 days, IQR: 94, 241 vs 191 days, IQR: 132, 257; [t‐test p‐value<0.001]), and preterm infants (34.6% preterm compared to 29.2% overall, χ^2^ = 26.7 p‐value<0.001).

**Table 1:** Bronchiolitis episodes, RSV tested episodes, and test results by ICD‐9‐CM diagnosis code, level of care, and bronchiolitis severity

By ICD‐9 code					
ICD‐9‐CM code	Total episodes N	Non‐tested episodes N (%)	Tested episodes N (%)	Test results	
				Negative N (%)	Positive N (%)
466.11	608	247 (40.6)	361 (59.4)	24 (6.7)	337 (93.3)
480.1	25	14 (56.0)	11 (44.0)	3 (27.3)	8 (72.7)
466.19	9,778	7,110 (72.7)	2,668 (27.3)	1,452 (54.4)	1,216 (45.6)
Total	10,411	7,371 (70.8)	3,040 (29.2)	1,479 (48.6)	1,561 (51.4)

By level of care (bronchiolitis severity)					
Level of care (bronchiolitis severity)	Total episodes N	Non‐tested episodes N (%)	Tested episodes N (%)	Test results	
				Negative N (%)	Positive N (%)
OPV	9,269	6,946 (74.9)	2,323 (25.1)	1,198 (51.6)	1,125 (48.4)
EDV	425	300 (70.6)	125 (29.4)	60 (48.0)	65 (52.0)
Hospitalization	717	125 (17.4)	592 (82.6)	221 (37.3)	371 (62.7)
Total	10,411	7,371 (70.8)	3,040 (29.2)	1,479 (48.7)	1,561 (51.3)

OPV = outpatient visit, EDV = Emergency department visit, and N = number

**Figure 1:**
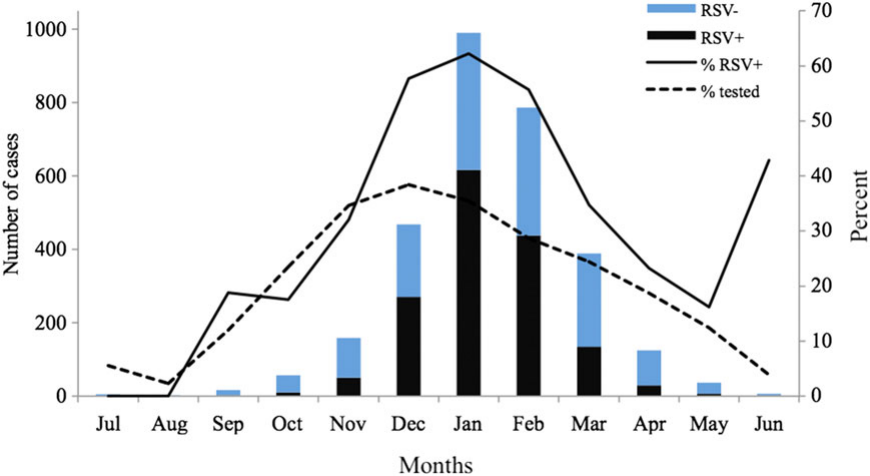
Distribution of infant bronchiolitis episodes with RSV testing and RSV positive bronchiolitis episodes among the PRIMA cohort subset of patients cared for by KPNC, 2006–2009. RSV+ is the number of RSV tested positive bronchiolitis episodes, RSV‐ is the number of tested RSV negative bronchiolitis episodes, %RSV+ is the percent of RSV positive bronchiolitis episodes among tested, and %tested is the percent of bronchiolitis episodes tested

**Figure 2:**
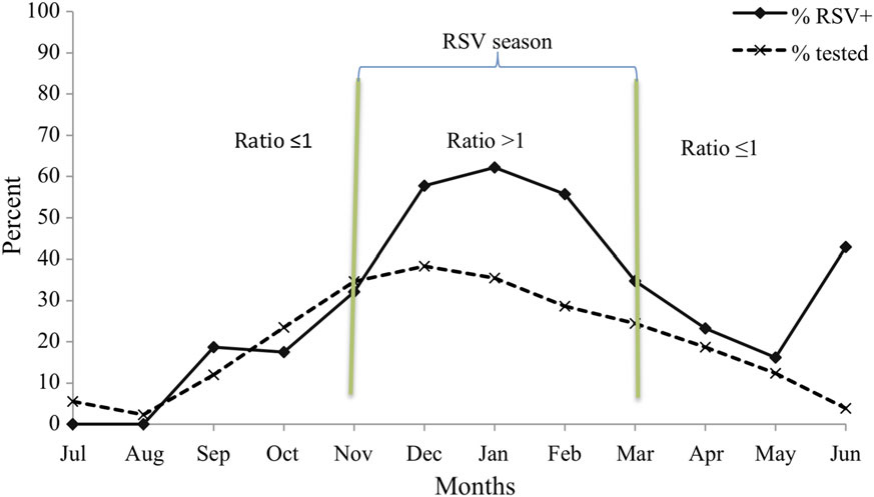
Monthly ratio of the proportion of RSV positive bronchiolitis episodes (%RSV+) to the proportion of tested bronchiolitis episodes (%tested)

Among 3,040 bronchiolitis episodes tested for RSV, 19.5% of episodes were hospitalizations, 4.1% of episodes were EDV, and 76.4% of episodes were OPV. This is in contrast to 7,371 episodes that were not tested, among which 1.7% of episodes were hospitalizations, 4.1% of episodes were EDV, and 94.2% of episodes were OPV. Fifty four percent of RSV‐tested bronchiolitis episodes during infancy were positive. These included: 93.4% among RSV‐bronchiolitis coded episodes, 72.7% among RSV‐pneumonia coded episodes, and 45.6% among non‐RSV bronchiolitis coded episodes (Table 1). The proportion of RSV positive bronchiolitis episodes was highest between the months of December and February (Figure 1, please also see Figure S1 panel A‐D for trends by bronchiolitis ICD‐9 code). We noted an upswing in the proportion of RSV positive cases in June, however, the number of episodes was very small (*n* = 7), and the RSV positive episodes occurred only among hospitalized infants in a single year (2007). The monthly ratio of percentage RSV positive bronchiolitis episodes to the percentage of RSV tested bronchiolitis episodes exceeded one between November and March, and identifies the RSV season in each of the study years (Figure 2).

Among tested bronchiolitis episodes that occurred in infants (≤12 months), 54.4% were RSV positive. When we limited to bronchiolitis episodes occurring among infants ≤6 months and ≤ 3 months, 56.9% and 66.3% were RSV positive, respectively. When we stratified bronchiolitis episodes in infants ≤6 months by type of healthcare encounter, RSV positive bronchiolitis episodes were highest among hospitalized episodes (68.5%), compared with EDV (66.7%) and OPV (52.6%) (Table S2). Further analysis limiting the bronchiolitis episodes to those episodes which occurred between December and February and in infants ≤6 months, the proportions of RSV positive bronchiolitis episodes were 77.2% among hospitalized episodes, 78.3% among EDV episodes, and 60.9% among OPV episodes, respectively (Table 2).

**Table 2:** Distribution of RSV positive bronchiolitis episodes among all infants (≤12 months), those ≤6‐months, and those ≤3‐months of age, and by type of healthcare encounter at age ≤ 6‐months in the PRIMA‐KPNC study cohort, 2006–2009

Month	ICD‐9 codes captured episodes	By age range						By encounter type at age ≤ 6‐months					
		≤12 months		≤6‐months		≤3‐months		OPV		EDV		Hospitalization	
		n	%+	n	%+	n	%+	n	% +	n	%+	n	%+
Dec	1,252	470	60.0	246	65.0	86	70.9	194	60.8	3	100	49	79.6
Jan	2,854	781	65.8	405	69.4	237	67.5	291	64.9	12	83.3	102	80.4
Feb	2,663	542	58.5	283	59.4	223	60.5	207	55.6	8	62.5	68	70.6
Total	6,769	1,793	62.0	934	65.2	546	65.2	692	60.9	23	78.3	219	77.2

n = number tested, which is denominator for % RSV positive (%+). Note: the number does not add up to the total sample size because only three peak months are included in the table (please see Table S2 for full year results).

We next stratified RSV tested bronchiolitis episodes by established risk factors for RSV. A stratified analysis was conducted on bronchiolitis episodes occurring during the first 6‐months by gestational age, a known risk factor for RSV infection^14^, ^15^ and an important factor determining eligibility and receipt of RSV‐immunoprophylaxis.^16^ RSV positive bronchiolitis episodes were higher among term or later (53.4% [1,358/2,542]) infants compared with premature infants (40.8% [203/498]), although a slightly higher proportion of preterm infants were tested (34.6% [498/1,440], compared with 28.3% [2,542/8,971] of term infant bronchiolitis episodes which were tested for RSV).

We conducted a multivariable logistic regression for outcome of RSV positive bronchiolitis episodes among tested episodes (Table 3). Younger age (in months) at the episode (adjusted odd ratio [aOR] = 1.03, 95% confidence interval [CI] = 1.01, 1.05) was modestly associated with RSV positive episodes. Hospitalization was associated with increased relative odds of RSV positive bronchiolitis episode compared with an OPV bronchiolitis episode (aOR = 2.11, 95% CI = 1.72, 2.59). No statistically significant association between EDV and RSV bronchiolitis episode was observed (EDV: aOR = 1.46, 95% CI = 0.99, 2.15). RSV tested bronchiolitis episodes occurring between December and February were positively associated with being RSV positive compared with those occurring during other months of the year (aOR = 3.49, 95% CI = 2.92, 4.18). In contrast, infants meeting AAP defined eligibility for RSV‐immunoprophylaxis guidelines (aOR = 0.39, 95% CI = 0.21, 0.71) or infants who ever received RSV‐immunoprophylaxis (aOR = 0.43, 95% CI = 0.25, 0.74) had decreased relative odds for an RSV positive bronchiolitis episode, compared with those who were ineligible and those who never received, respectively.

**Table 3:** Factors associated with infant positive RSV test outcome (*N* = 3,040)

Variable	OR (95% CI)	aOR (95% CI)
Age at bronchiolitis	0.998 (0.998, 0.999)	0.999 (0.998, 1.000)
Infant sex		
Male	0.888 (0.767, 1.027)	0.880 (0.754, 1.026)
Female	Reference	Reference
Bronchiolitis severity		
EDV	1.154 (0.805, 1.654)	1.463 (0.997, 2.147)
Hospitalization	1.788 (1.485, 2.152)	2.109 (1.717, 2.591)
OPV	Reference	Reference
RSV peak months		
December to February	3.419 (2.869, 4.072)	3.492 (2.916, 4.182)
March to November	Reference	Reference
Eligibility for RSV prophylaxis		
Yes	0.222 (0.141, 0.349)	0.385 (0.208, 0.713)
No	Reference	Reference
RSV prophylaxis receipt		
Yes	0.272 (0.185, 0.399)	0.431 (0.252, 0.737)
No	Reference	Reference

aOR = adjusted (multivariable) odds ratio, OR = Odds ratio, and CI = Confidence interval

In addition to evaluating factors that were associated with testing for RSV, we conducted multivariable logistic regression including infant age at diagnosis, meeting AAP defined eligibility for RSV‐immunoprophylaxis guidelines, level of care, calendar month during which illness occurred, infant sex, and parent reported infant race as covariates (Table 4). The relative odds of RSV testing slightly decreased in older age infants (aOR = 0.99, 95%CI = 0.99, 0.99), but increased in infants meeting AAP defined eligibility for RSV‐immunoprophylaxis (aOR = 1.65, 95% CI = 1.28, 2.13), among infants hospitalized for bronchiolitis (aOR = 12.96, 95% CI = 10.54, 15.94), and during the months from October (aOR = 4.92, 95% CI = 1.86, 13.00) to April (aOR = 3.80, 95% CI = 1.47, 9.79), compared to July. The month in which it was most likely for a bronchiolitis episodes to be tested for RSV was December (aOR = 10.79, 95%CI = 4.26, 27.35). It should be noted, however, that while we have provided this information, it cannot address whether RSV testing influenced hospitalization.

**Table 4:** Factors associated with RSV testing (*n* = 10,410)

	OR (95% CI)	aOR (95% CI)
Age	0.99 (0.99, 0.99)	0.99 (0.99, 0.99)
Race		
White	0.98 (0.89, 1.07)	0.95 (0.86, 1.04)
All non‐white	Reference	Reference
Sex		
Female	1.04 (0.95, 1.13)	1.01 (0.92, 1.10)
Male	Reference	Reference
RSV prophylaxis eligibility		
Yes	1.59 (1.26, 2.01)	1.65 (1.28, 2.13)
No	Reference	Reference
Bronchiolitis severity		
EDV	1.25 (1.01, 1.54)	1.31 (1.05, 1.63)
Hospitalization	14.16 (11.61, 17.27)	12.96 (10.54, 15.94)
OPV	Reference	Reference
Test month		
January	9.46 (3.83, 23.39)	9.08 (3.60, 22.93)
February	6.92 (2.79, 17.12)	6.43 (2.55, 16.26)
March	5.54 (2.23, 13.76)	5.21 (2.05, 13.20)
April	3.95 (1.57, 9.96)	3.80 (1.47, 9.79)
May	2.43 (0.92, 6.39)	2.46 (0.91, 6.62)
June	0.71 (0.21, 2.39)	0.58 (0.17, 2.04)
July	Reference	Reference
August	0.41 (.077, 2.17)	0.41 (0.07, 2.24)
September	2.34 (0.83, 6.58)	2.28 (0.79, 6.58)
October	5.27 (2.05, 13.58)	4.92 (1.86, 13.00)
November	9.10 (3.63, 22.85)	8.88 (3.46, 22.79)
December	10.73 (4.32, 26.63)	10.79 (4.26, 27.35)

aOR = adjusted (multivariable) odds ratio, OR = Odds ratio, and CI = Confidence interval

## 4 DISCUSSION

In a large population‐based study of infants who were continuously enrolled in an integrated healthcare system, we calculated the proportion of ICD‐9 defined and laboratory tested bronchiolitis episodes that were attributable to RSV. ICD‐9 coded bronchiolitis episodes occurring during the peak months of winter viral season, among younger infants, and among those with higher levels of care, were more likely to be attributable to RSV.

In agreement with other studies,^7^, ^17^ the majority of ICD‐9 coded and RSV tested bronchiolitis episodes during infancy were associated with RSV infection (51.4%). Our study confirms that the proportion of RSV positive bronchiolitis episodes is higher (73.6% of RSV tested bronchiolitis episodes) during the peak months of winter viral season (December to February), among infants under 6 months of age, and among those hospitalized for bronchiolitis. However, the proportion of bronchiolitis episodes that were tested for RSV was also higher between the months of December and February. The monthly ratio of the proportion of RSV positive bronchiolitis episodes to the proportion of RSV ever tested bronchiolitis episodes exceeded one between November and March, and was less than or close to one the remainder of the year (Figure 2). However, testing in our study is biased by well recognized RSV risk factors or clinical presentations, such as younger age, more severe clinical disease, and prematurity, among others factors. Many of these factors are also more likely to be associated with RSV than other viral etiologies of respiratory illness in infancy. These pretest features inform clinical testing and, thus, pretest probability of having RSV, which increases the specificity of use of electronic record coding for RSV bronchiolitis for purposes of RSV surveillance.

In a multi‐year surveillance study of 31 US ED sites, Makari and colleagues reported that 62% of ICD coded bronchiolitis EDV encounters were RSV positive by PCR during the peak period (January 15 to end of February).^18^ Hall and colleagues also reported in a multi‐year prospective study that 26% of acute respiratory illnesses in children age < 24 months and hospitalized during October through March were attributable to RSV.^19^ The fact that the proportion of RSV positive bronchiolitis episodes in EDV and hospitalization were similar in our analysis shows that results from a prospective study by Makari and colleagues, which calculates proportion of RSV positive bronchiolitis in EDV, could be applied to calculate the proportion of RSV positive among infants hospitalized for bronchiolitis coded episodes. However, both studies are substantially different from our study. Most notably, Hall and colleagues included children up to age 2 years and those who met criteria for upper and lower respiratory illness. Makari and colleagues reported EDV encounters only, and the reported peak period was restricted to one month (but included two‐month shoulder periods on each side). Our study is neither randomized, prospective, nor one in which standardized RSV testing was done. We recognize that the testing bias hinders generalizability of our results, however, it is likely representative of what happens across multiple acute care settings (outpatient, emergency department, and in‐patient hospitalization) compared to those studies conducted solely in emergency department settings. In addition, our study reflects real world testing practice conducted in clinical/healthcare setting in which electronic medical record data for RSV surveillance can be used.

The majority of the infants in our study were deemed to be low‐risk (97%), born at term or later, and without underlying conditions. Three percent of episodes were among those born preterm, and RSV‐immunoprophylaxis eligible (high‐risk) infants who may or may not have received RSV‐immunoprophylaxis. In our multivariable regression analysis, there were decreased relative odds of having at least one RSV positive bronchiolitis episode for RSV immunoprophylaxis eligible vs. non‐eligible and for subjects receiving RSV‐immunoprophylaxis. Eighteen percent of those eligible to receive RSV immunoprophylaxis did not receive any RSV‐immunoprophylaxis and 1% of those ineligible, received at least one dose of immunoprophylaxis. It may be that the routine care of high‐risk infants is different from the care for term or later birth infants. Parents may be less likely to send high‐risk infants to daycare, may take extra caution with regard to ill contacts, and healthcare providers may do more testing to include a larger proportion of infants with a broader spectrum of respiratory illness, as evidenced by the higher proportion tested (34.6% vs. 28.3%).

Our study has several strengths, including a large sample of bronchiolitis episodes which were tested for RSV. In addition, the majority of tests (78.3%) were done using the most sensitive clinical testing modality for RSV detection, PCR. Antigen and culture RSV detection results were used only when PCR was not performed. This approach overcomes the limitation of most surveillance studies, which are based on low sensitivity antigen RSV detection methods.

Despite the strengths mentioned above, this study has important limitations to consider. First, the criteria for ordering of RSV laboratory tests was not standardized and varied by level of healthcare episode (OPV, EDV, and hospitalization). Moreover, we do not know the proportion of RSV positive episodes among those 71% of non‐tested episodes. It is likely that the prevalence of RSV positive episodes would be lower if all ICD‐9 coded bronchiolitis episodes were tested. However, as shown in our regression results, tests were more commonly ordered for bronchiolitis episodes in younger infants, during peak months of winter viral season, and for infants with higher severity healthcare encounters. AAP guidelines^12^ do not recommend routine RSV testing, and recommend testing only in infants hospitalized while on RSV‐immunoprophylaxis. If testing was conducted at random and across the board, it is possible that the proportion of RSV positive bronchiolitis would be lower, as testing would also include those with a lower likelihood of having RSV. As the objective of our study was to determine the proportion of clinically‐coded bronchiolitis events that are RSV positive among bronchiolitis episodes that were tested for RSV, our results are not generalizable to all bronchiolitis coded events. Second, a test for RSV is more likely to be negative if infants present further into their illness when viral shedding is diminished and among infants with less severe disease. RSV viral shedding in infants is 3–10 days,^20^ with severe cases shedding longer, and because most RSV bronchiolitis episodes present with fever when they are still shedding virus, it is less likely that RSV positive bronchiolitis episodes would be missed by PCR testing compared to those with milder illness. Lastly, this is a study conducted in one region of the US and included a single privately insured healthcare system, and, thus, results from our study may not be generalizable to other populations. However, the market share for KPNC is about 44 percent, composed of commercial coverage (87%) and Medicare (11%), and the KPNC members are similar demographically to the Northern California population.^21^ Although KPNC is not representative of the overall US population, our findings provide important information about the use of bronchiolitis ICD‐9 codes in infants during RSV season for estimating RSV related bronchiolitis episodes.

## 5 CONCLUSION

Estimates of RSV‐bronchiolitis burden and seasonal distribution are often based on ICD‐9 codes for bronchiolitis. We provide estimates of ICD‐9 codes for bronchiolitis being attributable to RSV based on results confirmed using RSV laboratory testing. The proportion of RSV positive bronchiolitis episodes varies by infant age at the illness, level of health care service used, and time of the year of the episode. Estimation of RSV positive bronchiolitis based on ICD‐9 codes can be improved by restricting to episodes occurring during peak months of winter viral season, among infants ≤6‐months age at time of illness, and among those requiring higher levels of healthcare. Strategies to improve the specificity of bronchiolitis‐coded episodes have implications for public health surveillance, the design of prevention strategies, and for research. As an example, identifying RSV peak season and target age group may help to efficiently channel scarce public health resources, as well as to implement prevention measures such as daycare closures or the testing of targeted prevention measures, to prevent disease spread. In addition, this information may inform the start and end of use of RSV immunoprophylaxis in high risk populations, improving prevention during periods of highest risk of infection, and potentially shortening duration, and, thus, cost of RSV immunoprophylaxis. Lastly, among those tested, the majority of RSV positive bronchiolitis episodes were among infants born at term or later gestational age, reinforcing that the greatest disease burden is among term infants.

## ETHICS APPROVAL AND CONSENT TO PARTICIPATE

This study was approved by both the Kaiser Permanente Northern California (KPNC) and Vanderbilt University Institutional Review Boards.

## FUNDING

This work was supported in part by National Institute of Health (NIH) grants: K24 AI 77930 (Hartert), R01 HS018454 (Hartert), 5T32HL087738 (Turi).

## COMPETING INTERESTS

Dr. Kedir N Turi, Dr. Pingsheng Wu, Dr. Kecia N Carroll, Ms. Tan Ding, and Ms. Tebeb Gebretsadik have no conflicts of interest. Dr. Tina V. Hartert served as a consultant to Medimmune through a Vanderbilt University School of Medicine contract to define respiratory outcomes for clinical and vaccine trials. Consultant fees were paid to Vanderbilt. She has also served as a consultant to Novavax and Regeneron. Dr. Gabriel Escobar, Ms. Eileen Walsh, and Ms. Sherian Xu Li have received grant support from Medimmune, LLC and Astra Zeneca to conduct research on RSV infections in infancy.

## AUTHOR CONTRIBUTIONS

Conceptualization: Pingsheng Wu, Tina V. Hartert, Gabriel J. Escobar

Funding Acquisition: Tina V. Hartert

Investigation: Pingsheng Wu, Tina V. Hartert, Kedir N. Turi

Methodology: Pingsheng Wu, Tina V. Hartert, Tebeb Gebretsadik, Tan Ding, Kedir N. Turi

Resources: Tina V. Hartert

Supervision: Tina V. Hartert

Writing – Original Draft Preparation: Kedir N. Turi

Writing‐Review & Editing: Pingsheng Wu, Tina V. Hartert, Tebeb Gebretsadik, Gabriel J. Escobar

## Supporting information


**Figure S1:** **Panel A**. Total ICD‐9 coded bronchiolitis episodes and RSV positive episodes by month. **Panel B.** Acute RSV bronchiolitis (ICD‐9 code = 466.11) episodes and RSV positive episodes by month. **Panel C.** Viral pneumonia (ICD‐9 code = 480.1) episodes and RSV positive episodes by month. **Panel D.** Acute bronchiolitis due to other infectious organisms (ICD‐9 code = 466.19) episodes and RSV positive episodes by month.

Table S1. ICD‐9‐CM Diagnosis Code relevant to the diagnosis of bronchiolitis as captured from KPNC electronic records.
Table S2. Distribution of RSV positive bronchiolitis episodes among all infants (≤12 months), those ≤6‐months, and those ≤3‐months of age, and by type of healthcare encounter at age ≤ 6‐months in the PRIMA‐KPNC study cohort, 2006–2009
