# Supplementary material for: Prevalence of infant bronchiolitis‐coded healthcare encounters attributable to RSV
Source: Health Sci Rep. 2018 Oct 12;1(12):e91. doi: 10.1002/hsr2.91 (PMC6295609; doi:10.1002/hsr2.91)
Supplement: Supplementary file 1 — Figure S1: Panel A. Total ICD‐9 coded bronchiolitis episodes and RSV positive episodes by month. Panel B. Acute RSV bronchiolitis (ICD‐9 code = 466.11) episodes and RSV positive episodes by month. Panel C. Viral pneumonia (ICD‐9 code = 480.1) episodes and RSV positive episodes by month. Panel D. Acute bronchiolitis due to other infectious organisms (ICD‐9 code = 466.19) episodes and RSV positive episodes by month. [file HSR2-1-e91-s001.pdf]

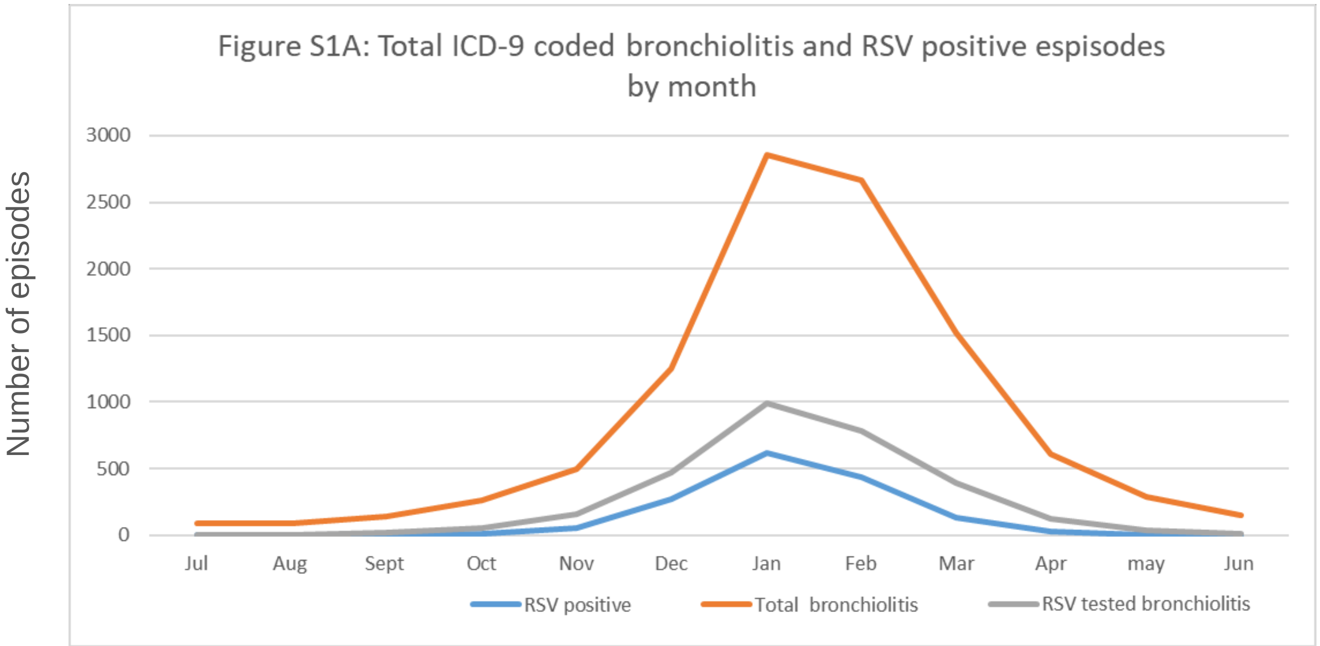
Figure S1A: Total ICD-9 coded bronchiolitis and RSV positive espisodes by month
Number of episodes
3000
2500
2000
1500
1000
500
0
Jul
Aug
Sept
Oct
Nov
Dec
Jan
Feb
Mar
Apr
may
Jun
RSV positive
Total bronchiolitis
RSV tested bronchiolitis

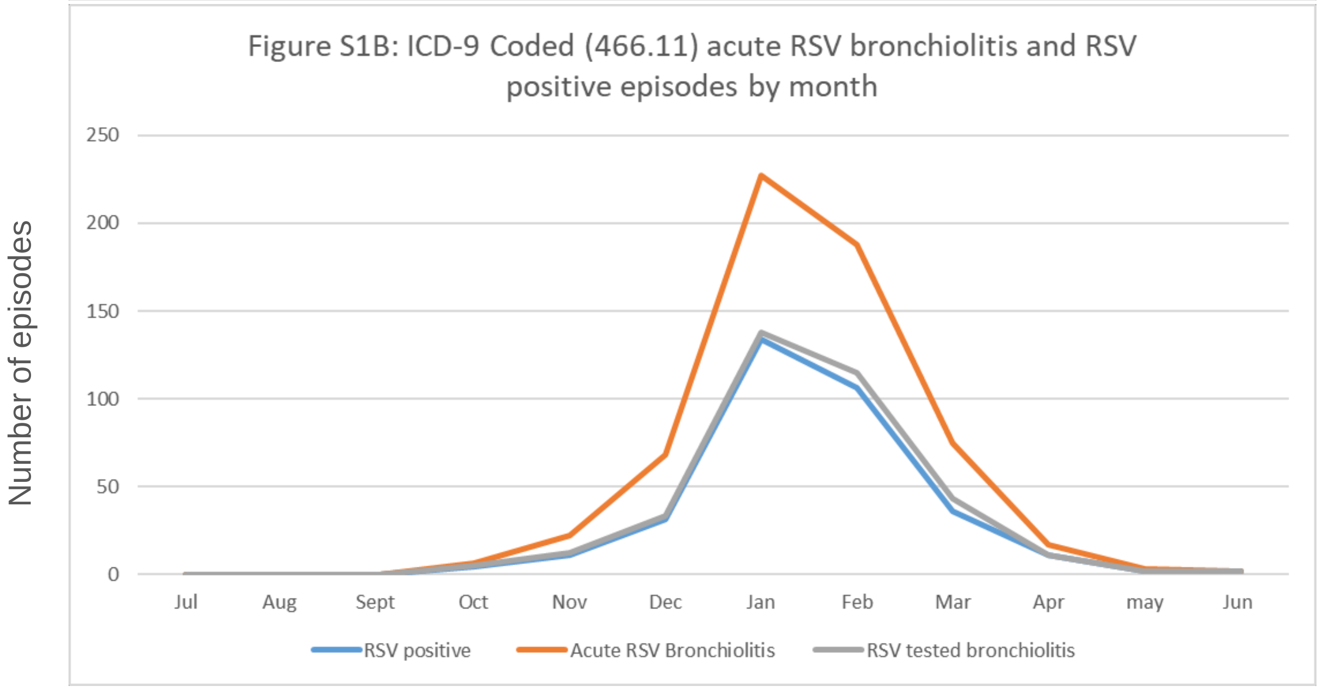
Figure S1B: ICD-9 Coded (466.11) acute RSV bronchiolitis and RSV positive episodes by month
Number of episodes
250
200
150
100
50
0
Jul
Aug
Sept
Oct
Nov
Dec
Jan
Feb
Mar
Apr
may
Jun
RSV positive
Acute RSV Bronchiolitis
RSV tested bronchiolitis

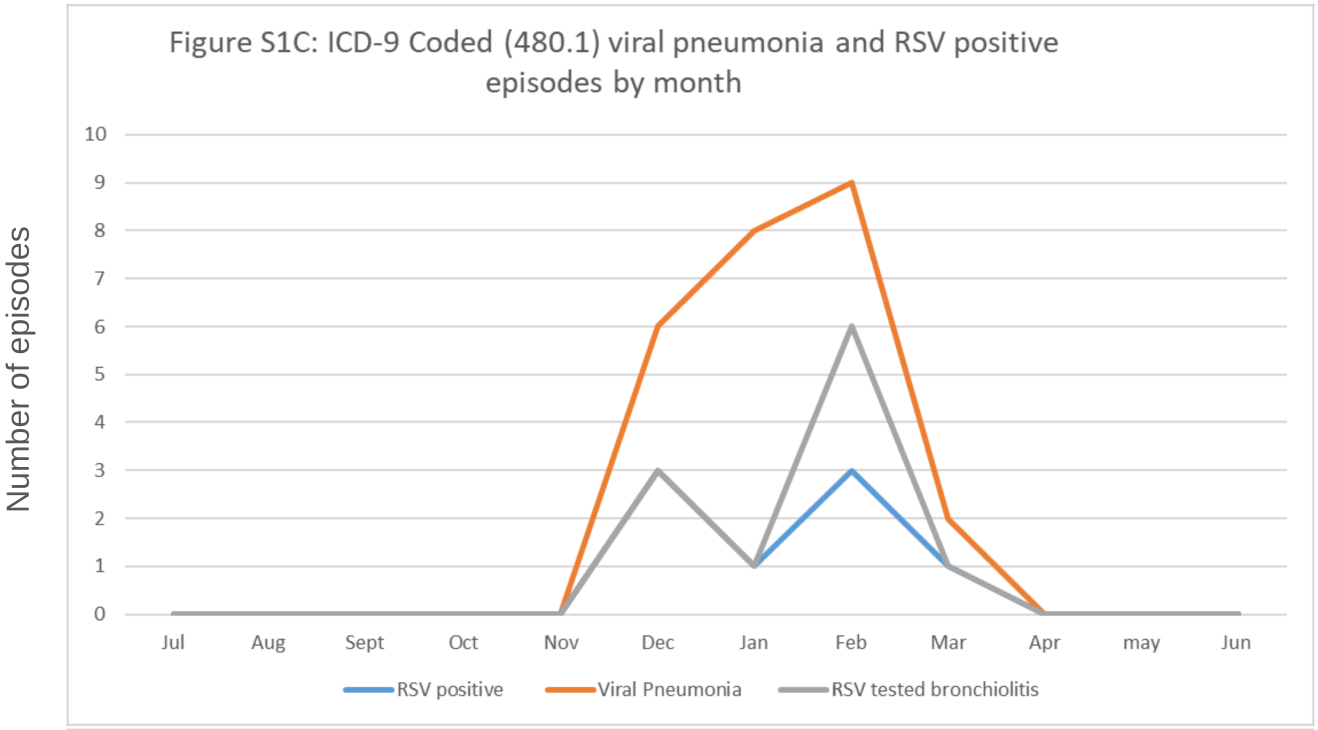
Figure S1C: ICD-9 Coded (480.1) viral pneumonia and RSV positive episodes by month
Number of episodes
10
9
8
7
6
5
4
3
2
1
0
Jul
Aug
Sept
Oct
Nov
Dec
Jan
Feb
Mar
Apr
may
Jun
RSV positive
Viral Pneumonia
RSV tested bronchiolitis

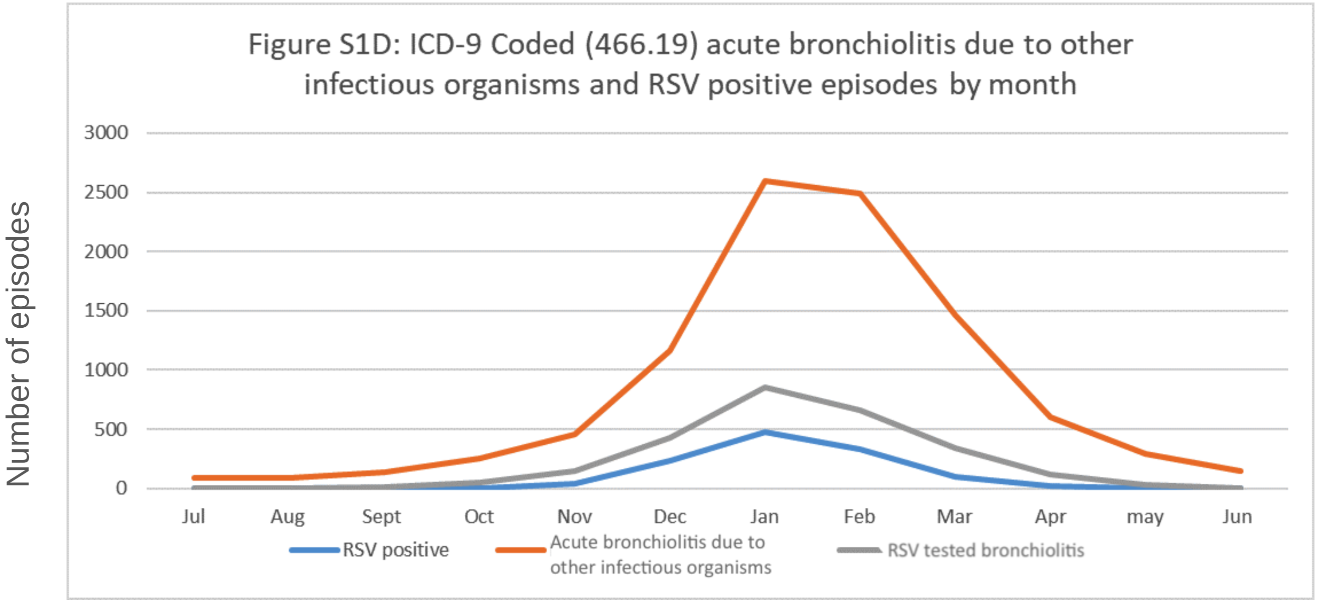
Figure S1D: ICD-9 Coded (466.19) acute bronchiolitis due to other infectious organisms and RSV positive episodes by month
Number of episodes
3000
2500
2000
1500
1000
500
0
Jul
Aug
Sept
Oct
Nov
Dec
Jan
Feb
Mar
Apr
may
Jun
RSV positive
Acute bronchiolitis due to other infectious organisms
RSV tested bronchiolitis
