# Supplementary material for: Prevalence of infant bronchiolitis‐coded healthcare encounters attributable to RSV
Source: Health Sci Rep. 2018 Oct 12;1(12):e91. doi: 10.1002/hsr2.91 (PMC6295609; doi:10.1002/hsr2.91)
Supplement: Supplementary file 2 — Table S1. ICD‐9‐CM Diagnosis Code relevant to the diagnosis of bronchiolitis as captured from KPNC electronic records. Table S2. Distribution of RSV positive bronchiolitis episodes among all infants (≤12 months), those ≤6‐months, and those ≤3‐months of age, and by type of healthcare encounter at age ≤ 6‐months in the PRIMA‐KPNC study cohort, 2006–2009 [file HSR2-1-e91-s002.docx]

Table S1. ICD-9-CM Diagnosis Code relevant to the diagnosis of bronchiolitis as captured from KPNC electronic records.

| ICD-9-CM Diagnosis Code | Diagnosis description |
| --- | --- |
| 466.11 | Acute bronchiolitis due to respiratory syncytial virus (RSV) |
| 480.1 | Pneumonia due to respiratory syncytial virus |
| 466.19 | Acute bronchiolitis due to other infectious organisms |

Table S2. Distribution of RSV positive bronchiolitis episodes among all infants (≤12months), those ≤6-months, and those ≤3-months of age, and by type of healthcare encounter at age ≤6-months in the PRIMA-KPNC study cohort, 2006 -2009

| Month | ICD-9  captured | Age | | | | | | | | Encounter type at age ≤6-months | | | | | |
| --- | --- | --- | --- | --- | --- | --- | --- | --- | --- | --- | --- | --- | --- | --- | --- |
|  |  | ≤12 months | | | ≤6-months | | ≤3-months | | OPV | | | EDV | | Hospitalization | |
|  |  | n | %+ | n | | %+ | n | %+ | n | | % + | n | %+ | n | %+ |
| Jan | 2,854 | 781 | 65.8 | 405 | | 69.4 | 177 | 71.8 | 291 | | 64.9 | 12 | 83.3 | 102 | 80.4 |
| Feb | 2,663 | 542 | 58.5 | 283 | | 59.4 | 120 | 68.3 | 207 | | 55.6 | 8 | 62.5 | 68 | 70.6 |
| Mar | 1,518 | 266 | 34.6 | 157 | | 35.7 | 49 | 48.9 | 106 | | 32.1 | 9 | 55.6 | 42 | 46.5 |
| Apr | 605 | 84 | 25.0 | 50 | | 24.0 | 13 | 30.8 | 35 | | 14.3 | 2 | 50.0 | 13 | 46.2 |
| May | 281 | 17 | 11.8 | 6 | | 0.0 | 0 | 0.0 | 4 | | 0.0 | 1 | 0.0 | 1 | 0.0 |
| Jun | 152 | 1 | 0.0 | 0 | | 0.0 | 0 | 0.0 | 0 | | 0.0 | 0 | 0.0 | 0 | 0.0 |
| Jul | 91 | 0 | 0.0 | 0 | | 0.0 | 0 | 0.0 | 0 | | 0.0 | 0 | 0.0 | 0 | 0.0 |
| Aug | 86 | 1 | 0.0 | 0 | | 0.0 | 0 | 0.0 | 0 | | 0.0 | 0 | 0.0 | 0 | 0.0 |
| Sep | 142 | 5 | 0.0 | 0 | | 0.0 | 0 | 0.0 | 0 | | 0.0 | 0 | 0.0 | 0 | 0.0 |
| Oct | 260 | 51 | 19.6 | 31 | | 22.6 | 8 | 50.0 | 21 | | 9.5 | 0 | 0.0 | 9 | 55.6 |
| Nov | 497 | 163 | 35.6 | 87 | | 41.4 | 27 | 59.3 | 57 | | 31.6 | 3 | 66.7 | 27 | 59.3 |
| Dec | 1,252 | 470 | 60.0 | 246 | | 65.0 | 89 | 70.8 | 194 | | 60.8 | 3 | 100 | 49 | 79.6 |
| Total | 6,769 | 2,381 | 54.4 | 1,265 | | 56.9 | 483 | 66.3 | 915 | | 52.6 | 39 | 66.7 | 311 | 68.5 |
